# Twenty years of big plant genera

**DOI:** 10.1098/rspb.2024.0702

**Published:** 2024-05-29

**Authors:** Peter W. Moonlight, Ludwig Baldaszti, Domingos Cardoso, Alan Elliott, Tiina Särkinen, Sandra Knapp

**Affiliations:** ^1^ Botany, School of Natural Sciences, Trinity College Dublin, Dublin, Ireland; ^2^ Royal Botanic Garden Edinburgh, Edinburgh, UK; ^3^ School of Geosciences, University of Edinburgh, Edinburgh, UK; ^4^ Instituto de Pesquisas Jardim Botânico do Rio de Janeiro, Rio de Janeiro, Brazil; ^5^ Instituto de Biologia, Universidade Federal da Bahia, Salvador, Brazil; ^6^ Natural History Museum, London, UK

**Keywords:** biodiversity, botanical history, flowering plants, generic concepts, nomenclature, species numbers

## Abstract

In 2004, David Frodin published a landmark review of the history and concepts of big plant genera. Two decades of taxonomic activity have taken place since, coinciding with a revolution in phylogenetics and taxonomic bioinformatics. Here we use data from the World Flora Online (WFO) to provide an updated list of big (more than 500 species) and megadiverse (more than 1000 species) flowering plant genera and highlight changes since 2004. The number of big genera has increased from 57 to 86; today one of every four plant species is classified as a member of a big genus, with 14% in just 28 megadiverse genera. Most (71%) of the growth in big genera since 2000 is the result of new species description, not generic re-circumscription. More than 15% of all currently accepted flowering plant species described in the last two decades are in big genera, suggesting that groups previously considered intractable are now being actively studied taxonomically. Despite this rapid growth in big genera, they remain a significant yet understudied proportion of plant diversity. They represent a significant proportion of global plant diversity and should remain a priority not only for taxonomy but for understanding global diversity patterns and plant evolution in general.

## 1. Introduction

**Figure 1.**
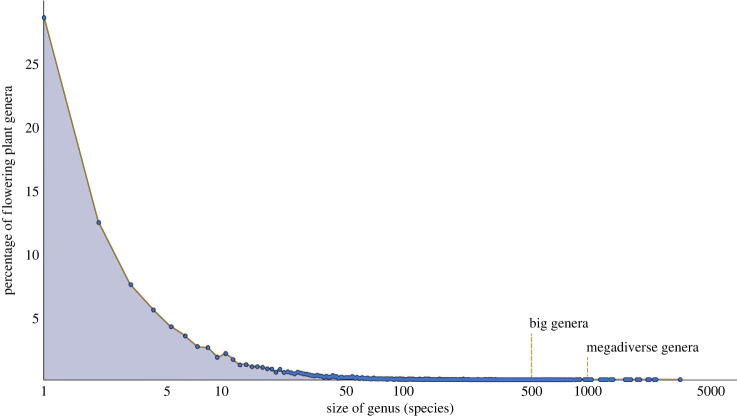
Size frequency of genera in angiosperms. Data from the December 2023 update of the World Flora Online (original data downloaded from IPNI and licensed CC BY are available from the NHM Data Portal at https://doi.org/10.5519/unb73250 [5]).

Species diversity in flowering plants is not evenly distributed. Diversity is highest in tropical latitudes, with hotspots for diversity in distinct regions within the tropical belt [1,2]. Sizes of taxonomic groups at all levels also vary across flowering plants. Some currently recognized [3] families such as Asteraceae and Orchidaceae have tens of thousands of species (more than 30 000 in both cases) while others consist of a single species (e.g. Amborellaceae, Cabombaceae). Genera also vary in size, more so today than in the early days of classification when genera were a way to memorize plant diversity [4]. The shape of the curve of genus size across flowering plants is highly skewed (figure 1) with 75% of flowering plant genera having five or fewer species. This highly skewed distribution of genus size is seen in animals as well, both in real-world and simulated data [6]. The existence of large genera of organisms is a distinctive pattern evident in the diversity of organisms [6,7] and not just the result of taxonomic splitting and/or lumping. Differing sizes of genera can be the result of many evolutionary processes; increased diversification, decreased extinction, and differing patterns of rate heterogeneity have all been suggested as drivers of taxon size [8]. Sigwart *et al*. [6] suggested that the extremely large genera of flowering plants and fungi [9] are likely incorrectly classified at the generic level. Several prominent cases of generic dismantling (e.g. *Cassia* [10]; *Schefflera* [11,12]; *Acacia* [13]) suggest this may be the case. Regardless of these concerns, many flowering plant genera are still large and are hypothesized to represent coherent, putatively monophyletic and diagnosable groups. The existence of comprehensive plant name indices and global taxonomic efforts in flowering plants make an assessment of the growth or shrinkage of big plant genera since the last review [14] possible.

Frodin [14] and Humphreys & Linder [4] have documented the history of generic concepts in flowering plants through time, discussing such aspects as changes in types of evidence and philosophies of classification. Both papers highlighted the differences between ‘splitters’ and ‘lumpers’ and showed that there have been trends through time in the acceptability of big genera. Linnaeus (from [15]) felt that no genus should have more than 100 species, while in the nineteenth century much larger genera were recognized by many authors, coinciding in part with emergence of evolutionary thinking. Humphreys & Linder [4] documented the same trends and showed that recognition of big genera was not purely the result of evolutionary thinking, but instead related to global synthesis. We do not repeat these historical analyses here, but instead refer readers to these publications where examples of different ways of treating plant genera are documented in detail.

Frodin [14] quoted E. J. H. Corner's [16, p. 100] disappointment that there was a ‘lack of study of big plant genera’, particularly in the tropics; Corner thought these groups would yield new evolutionary insights and that ignoring them was an impediment to understanding diversification, particularly in areas of high biodiversity. In Frodin's [14] review of big plant genera, he synthesized the impediments to study of these genera in the past and suggested that new techniques and methods would enable their study. Humphreys & Linder [4] showed that the advent of molecular data in classification has resulted in some genera becoming larger, compared to classification based on morphology alone, but the concept of monophyly has also been important. Stable generic names and concepts help orient identification, focus evolutionary hypotheses and enable predictability; ‘good’ genera are both stable and predictive, leading to unambiguity and acceptance [17]. Echoing Humphreys & Linder [3], Muñoz-Rodríguez *et al*. [18] suggested that ‘good’ genera should be monophyletic, diagnosable, and complete (i.e. contain within them all the species in the monophyletic group). An additional principle guiding generic classification brought out by Humphreys & Linder [4] was a general desire for stability; generic concepts that minimized taxonomic name changes are preferred.

Big plant genera are often portrayed as impenetrable and impossible to study [13], but it is now feasible to study big genera, not only due to new methods and new data types, but also due to the advent of global collaborations such as those spurred by the US National Science Foundation's ‘Planetary Biodiversity Inventories' (PBI) Program (https://www.nsf.gov/news/news_summ.jsp?cntn_id=103065), where groups of taxonomists were supported to work at a global level on monophyletic groups. Three of the funded PBI projects were undertaken on big genera of flowering plants (*Solanum* [19]; *Euphorbia* [20]; *Miconia* [21]) spurring the creation or strengthening of global consortia. Other global consortia have also undertaken work with large genera such as sedges (*Carex* [22]), sages (*Salvia* [23]), begonias (*Begonia* [24]) and morning glories (*Ipomoea* [25]). These global collaborations provided exactly the conditions Bentham [26] suggested were needed for real understanding of plant diversity almost 250 years ago.

Taxonomists today are more connected than at any time in the past and are working together in the description and documentation of plant diversity [27]. No longer are single taxonomists developing hypotheses of new taxa or the criteria for delimiting them, but synthesis and consensus are common. Given that new species of flowering plants are being described at a constant rate of approximately 2000 per year [28], it is expected that all genera of flowering plants will have become larger in the last decades. Big genera are stated to contain a significant proportion of diversity [18], but since Frodin [14] no attempt has been made to document what this proportion is or track how it has changed.

Twenty years on from Frodin's [14] highly impactful paper on the identity and importance of big plant genera we assess the identity and growth of plant genera with more than 500 species today (end of 2023). We use data from emerging global compilations of taxonomic study to ask: (1) Are the big plant genera identified by Frodin [14] still big? (2) Have big genera grown since Frodin [14] or have these big genera shrunk? (3) Is there a broad geographical pattern to genus size? (4) What factors (re-circumscription or new description) drive changes in genus size? (5) Do these factors differ in different-sized genera?

## 2. Material and methods

### (a) Big plant genera

The list of big angiosperm genera with more than 500 species (infraspecific categories not included) provided by Frodin [14] was based largely upon the World Checklist of Seed Plants (WCSP) published from 1995 [29–31] with gaps filled in by reference to the second edition of the *Plant Book* [32]. The WCSP evolved to become the World Checklist of Vascular Plants (WCVP) [33]. For our analyses we used World Flora Online (WFO) [34] to identify big plant genera based on their most recent update (https://wfoplantlist.org/, accessed 30 December 2023). WFO is based on a wide and inclusive global network of taxonomic contributors who curate names via Taxonomic Expert Networks (TENs) [33] and uses data from the WCVP [33] (evolved from WCSP) for groups lacking TENs. Like Frodin [14] we only used the taxonomic rank of species, we did not include infraspecific taxa or unresolved names (those whose status is uncertain).

We based the size of a genus on the number of accepted names (equivalent to valid names in the *International Code of Zoological Nomenclature* [35]). An accepted name is one that is considered the correct name for a species; every accepted name can have several to many synonyms (other names published that in the opinion of the expert belong to the same species). We calculated the ratio of synonyms to accepted names based on data from WFO.

### (b) Geographical patterns in big genera

To compare Frodin's [14] analysis with ours we used a broad definition of geography, following his methodology but updating to current knowledge. We categorized genera geographically as either tropical or temperate based on their centre of diversity according to the latest edition of the *Plant Book* [36] following Frodin [14]. Additionally, whenever a genus was described as either ‘cosmopolitan’ or ‘sub-cosmopolitan’ in the *Plant Book* or had both temperate and tropical diversity centres, we classified it as globally diverse. Genera primarily found in temperate regions but also occurring in tropical montane systems were coded as temperate.

### (c) New names in big genera

To assess reasons behind growth of big genera since Frodin [14], we quantified the relative contributions of taxonomic novelties and generic consolidation (recombination) to the growth of big plant genera. We accessed a list of species names that are treated as accepted by the WFO and have been published since 1 January 2000. To classify these names, we downloaded data for all new species names published since 1 January 2000 from the International Plant Names Index [36] (accessed 12 December 2023). In accordance with the rules of the *International Code of Nomenclature for algae, fungi, and plants* [37] IPNI classifies new names as: (i) tax. nov. (new taxon); (ii) comb. nov. (new combination); (iii) comb. nov. et stat. nov. (new combination at a new rank); (iv) nom. nov. (replacement name); (v) nom. nov. et stat. nov. (replacement name at a new rank); (vi) stat. nov. (name at new rank); or as (vii) unknown. We term names in category (i) as ‘new species’, names in categories (ii)–(vi) as ‘recombined species’, and names in category (vii) as ‘unknown’. We also classified names treated as accepted in WFO but missing from IPNI as ‘unknown’. To minimize the number of names classified as unknown, we manually assessed 120 names published in the genus *Stelis* in 2001 or 2002 that were classified as unknown; this accounted for most of the unknown names in big genera in the dataset.

To determine whether big and megadiverse plant genera have grown as expected given their size, we calculated the size of all genera based on WFO. We then removed all accepted species described this millennium from this dataset (62 042 species). We then re-allocated these species to genera with the probability based upon genus size and repeated this 1000 times. We then tested whether our observed number of newly described species in big (86 058 species) and megadiverse (46 798 species) genera fell within the predicted distribution of the number of species in those genera.

We were not able to assess reasons for generic shrinkage using our data set. Genera can become smaller because of synonymy (species accepted in the past that are not accepted now) or because species are no longer thought to belong and are transferred to another genus. IPNI does not record taxonomic usage (synonymy), and until recently did not record the previously published name on which a new combination or name at new rank is based (basionym or replacement name; see [37]). Names published in a genus, moved to another and then moved back are similarly opaque to our analysis. We include synonymy rate for all big genera in table 2.

## 3. Results

### (a) Big and megadiverse plant genera in 2023

There are 86 big plant genera (with more than 500 species) that collectively include 86 058 species (electronic supplementary material, table S1), representing 25% of the 344 064 flowering plant species accepted in WFO [34]. Big flowering plant genera are found in 46 of the 418 currently recognized families (11%) in 22 of the 64 recognized orders of angiosperms. Only fifteen families each include more than one big plant genus (3.6%), the largest of these are Orchidaceae (10 genera), Asteraceae (8), Leguminosae (7), Myrtaceae (4), and Rubiaceae (4).

Only 28 big genera are megadiverse (with more than 1000 species; table 1). These megadiverse genera include 46 798 species, representing 13.6% of all flowering plant species. Megadiverse genera are distributed across 18 families (4.3%), with only the Orchidaceae (5 genera), Asteraceae (2), Leguminosae (2), Piperaceae (2), Euphorbiaceae (2), and Myrtaceae (2) including more than one megadiverse genus.

**Table 1.** Megadiverse (more than 1000 species) flowering plant genera, showing changes from 2004.

genus	family	Frodin 2004		this study				
		spp.	rank	spp.	rank	spp. change	rank change	geography
*Astragalus*	Leguminosae	3270	1	**3239**	1	↓31	=0	global
*Taraxacum*	Asteraceae	excluded [60, hundreds of microspecies]		**2387**	2	↑new	↑new	temperate
*Hieracium*	Asteraceae	excluded [90, *ca* 1000 microspecies]		**2349**	3	↑new	↑new	temperate
*Carex*	Cyperaceae	1795	5	**2328**	4	↑533	↑1	global
*Bulbophyllum*	Orchidaceae	2032	2	**2190**	5	↑158	↓3	tropical
*Piper*	Piperaceae	1055	14	**2169**	6	↑1114	↑8	tropical
*Euphorbia*	Euphorbiaceae	1836	4	**2157**	7	↑321	↓3	global
*Begonia*	Begoniaceae	1484	6	**2144**	8	↑660	↓2	tropical
*Miconia*	Melastomataceae	1000	17	**1939**	9	↑939	↑8	tropical
*Epidendrum*	Orchidaceae	800	27	**1872**	10	↑1072	↑17	tropical
*Rubus*	Rosaceae	excluded [250+, infinity of apomictic lines]		**1732**	11	↑new	↑new	global
*Senecio*	Asteraceae	1250	9	**1681**	12	↑431	↓3	global
*Psychotria*	Rubiaceae	1951	3	**1650**	13	↓301	↓10	tropical
*Dendrobium*	Orchidaceae	1371	7	**1647**	14	↑276	↑7	tropical
*Ranunculus*	Ranunculaceae	600	46	**1616**	15	↑1016	↑41	temperate
*Peperomia*	Piperaceae	1000	18	**1384**	16	↑384	↑2	tropical
*Anthurium*	Araceae	789	29	**1346**	17	↑557	↑12	tropical
*Stelis*	Orchidaceae	<500 [370]		**1338**	18	↑new	↑new	tropical
*Eugenia*	Myrtaceae	1113	13	**1284**	19	↑171	↓6	tropical
*Solanum*	Solanaceae	1250	10	**1238**	20	↓12	↓10	global
*Syzygium*	Myrtaceae	1041	16	**1236**	21	↑195	↓5	tropical
*Acacia*	Leguminosae	1353	8	**1234**	22	↓119	↓14	tropical
*Rhododendron*	Ericaceae	1000	19	**1195**	23	↑195	↓4	temperate
*Lepanthes*	Orchidaceae	800	28	**1189**	24	↑389	↑4	tropical
*Croton*	Euphorbiaceae	1223	11	**1178**	25	↓45	↓14	tropical
*Salvia*	Lamiaceae	945	20	**1049**	26	↑104	↓6	global
*Phyllanthus*	Phyllanthaceae	833	25	**1025**	27	↑192	↓2	tropical
*Impatiens*	Balsaminaceae	850	23	**1002**	28	↑152	↓5	tropical

Estimates of species numbers for those genera either excluded or not meeting Frodin's [14] 500 species threshold taken from Mabberley [32] and presented in square brackets. Data from 2023 are adapted from the World Flora Online December 2023 update. Up arrows indicate an increase in species numbers or rank, down arrows a decrease. For the complete list of big and megadiverse genera including synonymy rates for each, see electronic supplementary material, table S1.

**Figure 2.**
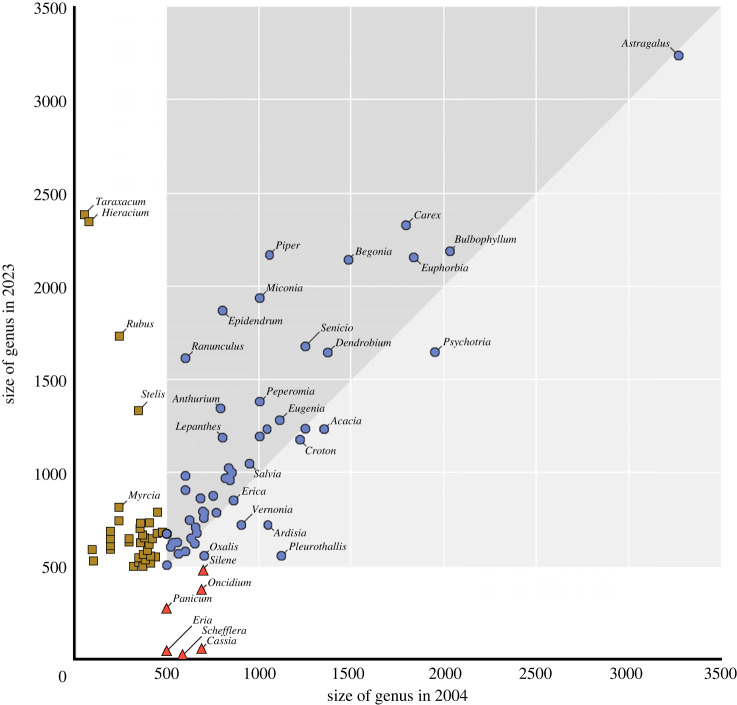
Size of flowering plant genera with more than 500 species in 2004 versus 2023. Data adapted from Frodin [14] and the World Flora Online (2023 update). Point colour and shape denote change of status between surveys: blue circles, present in both lists; red triangles, lost from the list in 2023; brown squares, new to the list in 2023. For the complete list of big and megadiverse genera, see electronic supplementary material, table S1.

Of the 57 plant genera Frodin [14] classified as big, only six now fall below the 500 species mark (*Silene*, Caryophyllaceae, 486 spp.; *Cassia*, Leguminosae, 60 spp.; *Oncidium*, Orchidaceae, 374 spp.; *Schefflera*, Araliaceae, 14 spp.; *Panicum*, Poaceae, 272 spp.; *Eria*, Poaceae, 48 spp.) and a further 12 genera have fewer species now than in 2004 (figure 2). By contrast, there have been 35 genera added to the list of big genera since 2004 and 39 of Frodin's genera are now larger than in 2004; 27 of these have increased by more than 100 species and three (*Piper*, Piperaceae; *Epidendrum*, Orchidaceae; *Ranunculus*, Ranunculaceae) have increased by more than 1000 species (figure 2).

Frodin [14] did not use the term megadiverse for genera with more than 1000 species, but he included 19 genera in his list that met our definition of megadiversity. Two of these are no longer megadiverse (*Ardisia*, Myrsinaceae, 734 spp.; *Pleurothallis*, Orchidaceae, 555 spp.), and five are smaller than in 2004 but are still include more than 1000 species (table 1). By contrast, 11 genera have entered the list of big genera and 12 genera are larger now than in 2004 (electronic supplementary material, table S1). Synonymy ratios (number of synonyms to accepted names) vary widely in big genera, from almost 5 synonyms per accepted name in *Solanum* to a ratio of 0.1 in the orchid genus *Lepanthes* (electronic supplementary material, table S1).

The number of species in big genera (more than 500 species) has risen by a third (30.6%) over the study period from 53 056 [13] to 86 058 (electronic supplementary material, table S1). For megadiverse genera with more than 1000 species the rise in species numbers is even more dramatic: from 27 190 to 46 978, a rise of 19 608 species (72.1%). There has been a 6% rise in the mean number of species per big genus, and a 13% rise in mean species number for megadiverse genera (1431 to 1671 species).

### (b) The geography of big plant genera

**Figure 3.**
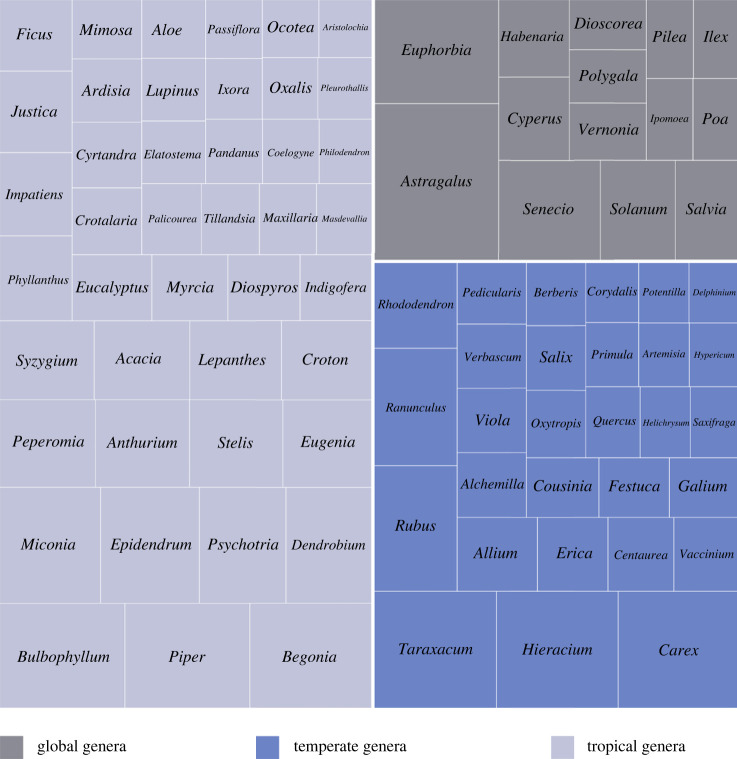
The broad geographical distribution of the big (more than 500 species) plant genera. Tropical-centred genera (pale blue, left-hand side of diagram); temperate-centred genera (dark blue, lower right-hand square); globally distributed genera (grey, upper right-hand square). Box size is proportional to genus size as recorded in WFO. Geographical distribution categories based on Mabberley [35]; data from the December 2023 update of the World Flora Online (WFO). For the complete list of big and megadiverse genera, see electronic supplementary material, table S1.

There is no clear geographical pattern in the occurrence of big genera using our broad categorization; big flowering plant genera are not exclusively tropical (figure 3). Of the 86 big genera, 44 (51.2%) occur mostly in the tropics whereas 27 (31.4%) are primarily from temperate regions. The remaining 15 (17.4%) genera are globally diverse with species in both tropical and temperate areas. The largest genus (*Astragalus*) is global, and the three largest genera with temperate distributions are those with a large proportion of apomictic or microspecies (e.g. *Taraxacum*, *Hieracium*, *Rubus*).

Megadiverse genera are present in each of the three broad distribution categories but show a slightly more pronounced geographical pattern biased towards the tropics, with species of 17 of the 28 megadiverse genera (60.7%) primarily occurring in tropical regions, while only four (14.3%) reach their highest diversity in temperate regions. Of the megadiverse genera, seven (25.0%) are globally species rich.

### (c) New names in big plant genera

A total of 68 297 species-level names have been published in the last twenty years (since 2000) across all flowering plants and are now treated as accepted in the WFO (table 2), accounting for 19.7% of all accepted species-level names. Of these, the majority are new species (52%) followed by new combinations (34.5%), with much smaller numbers of other categories of names.

**Table 2.** Number and type of species-level names in big flowering plant genera published since the year 2000 and treated as accepted in the December 2023 update of the World Flora Online (WFO).

	new species	recombined species	unknown	total
all genera	35 487 (55 110)	27 607 (57 965)	5203^a^ (1772)	68 297 (114 837)
big genera	10 618 (14 823)	4037 (5098)	222^a^ (195)	14 877 (14 877)
small genera	24 869 (40 287)	23 570 (52 867)	4981^a^ (1577)	53 420 (94 694)

The total number of species-level names published according to IPNI [38] is presented in parentheses. Classification of species names is adapted from IPNI as described in the text. Big genera are those with more than 500 species, small genera are those with fewer than 500 species.

^a^Names included in the WFO but not in IPNI are classified as ‘unknown’.

**Figure 4.**
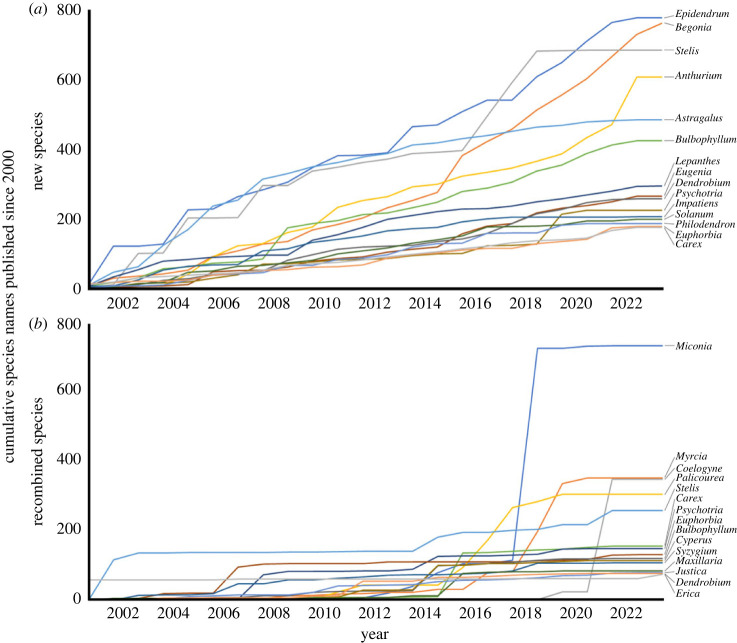
Cumulative publication of species-level names in big plant genera with more than 500 species since the year 2000. The top 15 genera for (*a*) new species and for (*b*) recombined species are shown. Only names treated as accepted in the December 2023 update of the World Flora Online are included. Data from IPNI and WFO.

Given the rate of new species descriptions this millennium [27], we would expect there to be an average of 86 770 newly described species (s.d.: 105.8 spp.) in big plant genera. Our observed value of 85 058 species is significantly fewer than expected (*p* ≤ 0.0001). By contrast, we would expect there to be an average of 45 774 species (s.d.: 80.6 spp.) in megadiverse genera, and our observed value of 46 798 species is significantly more than expected (*p* ≤ 0.0001). The numbers and sorts of names published in each big plant genus are shown in full in electronic supplementary material, table S2. Over the study period, new species were published and accepted in all 86 big genera (electronic supplementary material, table S2). The highest number of new species was published in *Epidendrum* and *Begonia*, followed by *Stelis*, *Anthurium*, *Astragalus* and *Bulbophyllum* (figure 4*a*). These six genera collectively account for 12.1% of accepted new angiosperm species published since 2000 and each genus accounts for more than 1% of this total. Only five genera have half of their currently accepted species names published since 2000: *Stelis*, *Coelogyne*, *Myrcia*, *Palicourea* and *Anthurium*.

Since 2000, 57 965 recombinations of previously published names have been published (table 2; electronic supplementary material, figure S1). Of these, 52 867 (91.2% of all recombinations) were in genera with fewer than 500 species. Accepted new combinations have been published in 70 of the 86 big genera (electronic supplementary material, table S2). The largest number were in *Miconia*, *Coelogyne*, *Palicourea*, and *Myrcia* (figure 4*b*), indicating that these genera have grown in a large part through generic lumping. These four genera collectively account for half of accepted new combinations published in big genera since 2000.

## 4. Discussion

Our study shows that a very small number of big flowering plant genera account for one in four of all known plant species. Even fewer megadiverse genera, each with more than 1000 species, account for 13% of all accepted flowering plant species. In the 20 years since the last review of big plant genera, the number and collective size of big genera has grown; we see a 66% increase in the number of genera comprising more than 500 species. Fewer species than expected were published in big genera, but newly described species were disproportionately concentrated within megadiverse plant genera. Most intriguingly, 14 of the most rapidly growing flowering plant genera account for 15% of all plant species described this millennium (figure 4*a*). These results suggest that while taxonomic revision of megadiverse genera is active, perhaps stimulated by funding for and global interest in these previously intractable groups, taxonomic effort in the smallest of the big genera (those with between 500 and 1000 species) is lagging behind.

What is evident is that the list of big genera as remained relatively stable over the past two decades, with only a few genera ‘split’ to the point that they no longer qualify as big. Geography too has remained stable, with big genera found in both tropical and temperate regions. Deployment of new tools in these large groups, coupled with the focus on monophyly and diagnosability as the principal criteria for taxon recognition [18] means that many of our big genera are likely to remain big. An increased focus on the criteria of monophyly and diagnosability means that future taxonomic work on the big genera not already under study will further refine these estimates in future reviews.

Since the year 2000, 73.2% of the species level names published in big genera have been new species, rather than names transferred from other genera. Species newly described in big genera since Frodin's [14] review comprise almost a third (30.7%) of all new species of flowering plants. This means big genera are not growing due to lumping, but due to the description of novelty. Due to their historical perceived intractability, big genera may be poorly known in comparison to plant genera with fewer species, leaving a sizeable proportion of species diversity to be discovered. Using vascular plant data from North America and Hawaii, Schwartz & Simberloff [39] showed that rare species are more common than expected in large lineages, the inverse of the pattern seen in birds and mammals. Most recently described plant species have small ranges [28], and studies in vertebrates have shown that newly described species are more at risk of extinction [40]. Several big genera are known for including large numbers of narrowly endemic species (e.g. *Astragalus*, *Miconia*, *Begonia*), perhaps leading to over-representation of big genera among recently described species. Several big plant genera (e.g. *Solanum*, *Carex*), however, are notable for their high number of widespread species.

Lack of global taxonomic revisionary work on big plant genera may also mean that fewer, recently described species have been correctly recognized as synonyms. The wide variation in synonymy rate across big genera (electronic supplementary material, table S1) may reflect significant biological differences among these genera, or on the other hand reflect global taxonomic effort [27,41]. Of newly described species published since 2010, 74.7% are accepted in big genera and 58.2% are accepted in genera with fewer than 500 species. If acceptance rates were equal to those in genera with fewer than 500 species, fewer than 1 in 4 of accepted newly described species published since 2000 would be in big genera. It is difficult to determine whether the relative lack of taxonomic effort in big plant genera leads to them being over- or underrepresented in the number of newly described species over our study period. Future work focused on exploring the effects of taxonomic effort on synonymy rate will be of interest to fully understand the variation in synonymy rates we see across big genera [42].

Our results demonstrate that over the last two decades the boundaries of big plant genera have remained much more stable than those of plant genera with fewer than 500 species. There were 2.6 times as many new combinations per species name in genera with fewer than 500 species than in big genera during our study period (table 2). These results may seem surprising given recent, well-publicized acts of generic consolidation (e.g. *Miconia* [43]; *Stelis* [44]). These examples will have garnered significant attention primarily because of the number of new combinations published in single works, but our results show that 7.8 times as many new combinations have been published in genera with fewer than 500 species than in big genera during our study period.

On the other hand, some of the new entries to the list of big genera (electronic supplementary material, table S1) are in the process of active study and they appear not to pass the key criteria of stability and monophyly (e.g. *Vaccinium* [45], *Ocotea* [46]), but have not yet been dismantled. Others of the big genera that were in Frodin's [14] list and remain big here (see electronic supplementary material, table S1) are the subject of ongoing discussion and differences of opinion as to those two key criteria (e.g. *Senecio* [47], *Psychotria* [48]). Nevertheless, these cases, like those cited above of generic consolidation, are in the minority and do not markedly disrupt the pattern of the big genera remaining big through time.

Big genera also can promote nomenclatural stability over the longer term by reducing the need for new combinations [24]. Although there are fewer currently accepted new combinations made in big genera, a larger percentage of new combinations made in big genera during our study period are now accepted than in genera with fewer than 500 species (82.7% versus 43.6%). Species in big genera appear to be subject to fewer unnecessary combinations that are later considered synonyms than are species in smaller genera (electronic supplementary material, figure S1). For example, imagine a genus with two species and one outgroup; here there is only one phylogenetic hypothesis that resolves the genus as monophyletic. Should this hypothesis be falsified, a generic re-circumscription is required. If any species in a fully sampled and monophyletic big genus is misplaced, i.e. if its position in a phylogeny with ≥500 nodes and one outgroup is incorrect, there are at least 498 other internal branches where it could be resolved without requiring generic re-circumscription. In short, it is less likely that a species is moved out of a big genus than a smaller genus. It is also possible that the number of generic re-circumscriptions in big genera may be lower because fewer phylogenetic treatments of big genera are published, and certainly because fewer fully sampled phylogenies of big genera are published [18].

While big genera may promote stability in generic circumscriptions, there is no reason to suspect that they promote stability in infrageneric species relationships. Recent phylogenetic studies within big plant genera have resulted in upheavals in formal (e.g. *Begonia* [24]) and informal (e.g. *Solanum* [49,50]) classifications within genera, while investigators in other groups have chosen not to apply infrageneric systems to describe all species relationships due to lack of completeness (e.g. *Ipomoea* [18]). Indeed, a lack of resources and available material mean that very few phylogenetic studies of big plant genera include comprehensive species coverage, or if they do, they may only do so with relatively few genetic markers. Consequently, our knowledge of the relationships of species in big plant genera may be less stable than our knowledge of the species relationships in plant genera with fewer than 500 species.

Monophyletic big genera should be diagnosable and thus easy to recognize, but species identification in big genera can be difficult. In our experience, herbarium cupboards are often filled with specimens identified only to genus level for many big genera, often limiting the utility of these valuable resources that represent nearly a quarter of all plant biodiversity for looking at local patterns of biodiversity or distributional processes. Better identification tools for these genera are needed to help non-experts navigate through their vast diversity. It is not enough just to know these genera are big, enabling others to use this knowledge is equally important. Confident identification of species in big genera is important to increase correct names on herbarium specimens in global data portals, as these data increasingly are used to explore distribution patterns at large spatial scales.

Predicting the future of individual big plant genera is tricky. The future of every big plant genus is dependent upon choices that rely upon taxonomic and phylogenetic knowledge. It is stochastic and unpredictable. But we can recognize trends. We predict that most of the big flowering plant genera identified here will remain big, and that the circumscription of big genera of flowering plants is likely to remain more stable than that of smaller genera. Our study supports the idea that big genera in flowering plants are stable and here to stay, and that species description in them is not levelling off. Big genera may represent an even larger proportion of flowering plant diversity by 2044.

## 5. Opportunities

Big flowering plant genera should no longer be seen as areas where in-depth scientific research and progress are difficult or impossible. New methods such as phylogenetics and recognition of the importance of monophyly and new techniques such as DNA sequencing and analytical morphology coupled with the trend to collaborative working mean that the sometimes daunting nature of large taxa can be overcome. Frodin's [14] impediments to growth of big plant genera are disappearing, thanks to molecular phylogenetics, digital resources and increased collaborative working across the globe. Gone are the days when a single taxonomist worked in isolation. Today's taxonomy, especially in big genera, is more productive when done collaboratively and in conjunction with scientists in other disciplines.

Big flowering plant genera offer unique opportunities for both taxonomists and evolutionary biologists. The existence of several large, monophyletic lineages with significant morphological disparity (*sensu* [9]) allows hypotheses about patterns and process to be tested in ‘replicate’, teasing out commonalities and differences. But not all big genera on our list have yet passed the test of monophyly and/or diagnosability, and these should be foci for the coalescence of international groups of taxonomists working globally, as Bentham [26] felt was necessary. The embedding of the WFO and its network of TENs [34] is a key step the taxonomic community should take if big genera are to step from the shadows of intractability. We list here a few of the evolutionary and ecological questions ripe for investigation in big genera; there are many more (e.g. [9]), and we are only limited by our imaginations.

— Do big genera represent adaptive radiations or lack of extinction?
— Do all big genera represent cases of synnovations [8] and confluences within lineages as has been shown for *Astragalus* [51] and to some extent in *Solanum* [52,53]?
— How do functional traits vary or covary within big genera? Does phenotypic plasticity play a role in diversification or life history in these species-rich lineages more or less than in smaller genera?
— Can controlling for phylogeny by using these big genera help to tease out processes that are lineage-specific versus more general characteristics in investigations of latitudinal gradients in functional traits (e.g. [54]) or diversity?
— Can finer-scale biome mapping uncover underlying patterns in temperate and tropical big genera that are not revealed using out broad characterization of geography?
— What are the diverse ways for flowering plant genera to be big? All big genera are clearly not the same, but how they differ is to date purely anecdotal.

Our reprise on Frodin's [14] seminal paper shows that big genera in flowering plants are still with us, they are growing, and due to advances in methods and technologies they no longer remain groups taxonomists fear to investigate. Big genera offer significant opportunities for global collaboration and interdisciplinary downstream study. They should be celebrated and worked on rather than feared.

## Data Availability

The data are provided on the Natural History Museum Data Portal (https://doi.org/10.5519/unb73250) [5] and in the electronic supplementary material [55].
